# Delineating the distinct role of AKT in mediating cell survival and proliferation induced by CD154 and IL-4/IL-21 in chronic lymphocytic leukemia

**DOI:** 10.18632/oncotarget.22292

**Published:** 2017-11-07

**Authors:** Elinor A. Chapman, Melanie Oates, Ishaque S. Mohammad, Barry R. Davies, Paul K. Stockman, Jianguo Zhuang, Andrew R. Pettitt

**Affiliations:** ^1^ Department of Molecular and Clinical Cancer Medicine, University of Liverpool, Liverpool, UK; ^2^ Oncology iMED, AstraZeneca, Cambridge, UK; ^3^ Royal Liverpool and Broadgreen University Hospitals NHS Trust, Liverpool, UK

**Keywords:** CLL, CD40 stimulation, AKT, proliferation, survival

## Abstract

The functional significance of AKT in chronic lymphocytic leukemia (CLL) remains unclear. Given the importance of non-malignant T cells in regulating clonal expansion in CLL, we investigated the role of AKT in T cell-mediated cytoprotection and proliferation using an established co-culture system in which primary CLL cells were incubated on a monolayer of transfected mouse fibroblasts expressing human CD40L (CD154). Stimulation of CLL cells via CD40 induced activation of AKT, which was closely associated with downregulation of its negative regulator PTEN, and protected CLL cells from killing by bendamustine. This cytoprotective effect of CD40 stimulation was prevented by a selective inhibitor of AKT. Stimulation of CLL cells with CD154 + IL-4 or IL-21 induced proliferation detected as reduced fluorescence of cells pre-stained with CFSE. AKT inhibition produced a significant, consistent reduction in proliferation induced by CD154 + IL-4 and a reduction in proliferation induced by CD154 + IL-21 in most but not all cases. In contrast, AKT inhibition had no effect on the proliferation of normal B cells induced by CD154 + IL-4 or IL-21. These findings indicate that AKT contributes in a significant way to T-cell mediated survival and proliferation signalling in CLL and support the clinical evaluation of AKT inhibitors in this disease.

## INTRODUCTION

Chronic lymphocytic leukemia (CLL) is a malignancy of CD5^+^ B lymphocytes that accumulate in the blood, bone marrow and secondary lymphoid tissues such as lymph nodes. It is the most common form of leukemia in adults in the Western countries [1]. Despite recent therapeutic advances involving purine analogues and monoclonal antibodies [2], the disease remains incurable, and new therapeutic agents are therefore urgently required.

Although the biology of CLL is still unclear [3, 4], disease pathogenesis is most likely influenced by microenvironmental factors in affected tissues. It is now accepted that chronic stimulation of the B-cell receptor (BCR) by antigen plays a key role in maintaining and expanding the malignant clone [5–7]. However, the survival and proliferation of CLL cells are also thought to depend on their interactions with accessory cells [8, 9]. One important interaction involves stimulation of CD40 on CLL cells by CD40 ligand (CD40L or CD154) on non-malignant T cells in affected lymph nodes [10, 11]. Such cross-talk between CLL cells and accessory cells has been increasingly recognized to play an important role in disease progression, most likely through the activation of certain pro-survival or mitotic signalling pathways.

One such pathway involves phosphatidylinositol 3-kinase (PI3K). The latter regulates a variety of important biological processes including cell growth, survival, migration, and metabolism [12, 13]. Particularly relevant to CLL is the fact that PI3K can be activated by diverse stimuli that include antigens, cytokines and chemokines, as well as adhesion molecules [14, 15]. Indeed, the aberrant activation of PI3K has been reported to contribute to the survival of CLL cells [16, 17]. Thus targeting the PI3K pathway becomes an attractive strategy for development of novel therapeutic agents. In fact, the novel PI3K p110δ isoform-specific inhibitor, idelalisib, has significant clinical activity in CLL and is now approved for this indication [18–20]. Mechanistically, idelalisib has been shown to exhibit multiple modes of action – directly decreasing cell viability and disrupting interactions that retain CLL cells in protective tissue microenvironments [21, 22], but also inducing egress of CLL cells from the lymph nodes [23].

The serine/threonine kinase AKT (also known as protein kinase B, PKB) is an important downstream effector of PI3K as it is largely responsible for PI3K-mediated pro-survival signalling [24, 25]. AKT is also involved in regulation of other cellular processes including angiogenesis, metabolism, growth/proliferation, protein synthesis and transcription [24, 26]. Dysregulation of AKT has thus been closely linked to the development of many human diseases including cancer [27, 28].

We and others have previously shown that AKT is critically involved in CLL cell-survival [29–32]. Pharmacological inhibition of AKT reduces the survival of CLL cells *ex vivo* under standard conditions [31, 33], indicating that AKT inhibitors may have therapeutic potential in CLL. However, given that the survival and proliferation of CLL cells is closely regulated by the CLL microenvironment, it is important to understand the effect of AKT inhibition in CLL cells that are exposed to relevant stimuli. To this end, we co-cultured primary CLL cells on a stromal monolayer of transfected mouse fibroblasts expressing human CD154 to mimic the lymph node microenvironment and explored the distinct effects of AKT in mediating the survival, growth and proliferation of CLL cells induced by CD40 stimulation.

## RESULTS

### Stimulation of CLL cells via CD40 induces AKT activation and reduced expression of PTEN irrespective of the presence of IL-4 or IL-21

**Figure 1:**
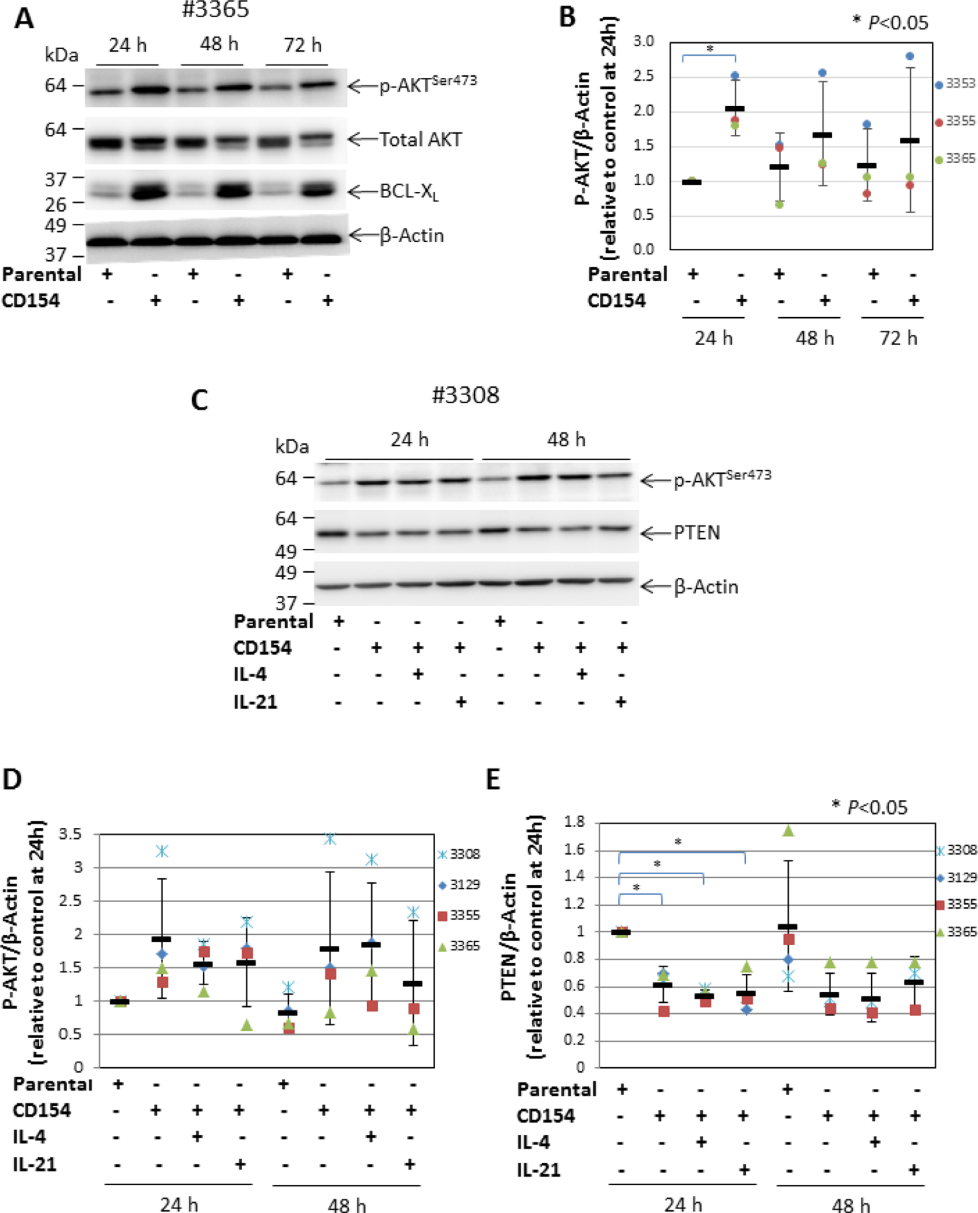
CD40 stimulation-induced AKT activation is associated with decreased expression of PTEN (**A**) CLL cells were cultured on a monolayer of parental control or CD154-expressing fibroblasts for 24, 48 and 72 h. At the indicated time points, CLL cells were harvested and analysed for the levels of p-AKT (serine 473) and total AKT by Western blotting. BCL-X_L_ was probed as a marker for CD40 stimulation. β-actin was used as a loading control for densitometric analysis. One representative blot from 3 CLL samples examined is shown. (**B**) shows a pooled data analysis of the effect of CD40 stimulation on levels of p-AKT in co-cultured CLL cells. In this and subsequent figures, each bar represents the mean ± SD, unless otherwise stated. (**C**) CLL cells were co-cultured for 24 and 48 h as in (A) but in the presence or absence of recombinant human IL-4 (10 ng/ml) or IL-21 (12.5 ng/ml). CLL cells were then harvested and analysed for levels of p-AKT (serine 473) and PTEN by Western blotting. One representative blot from 4 CLL samples examined is shown. (**D**) shows a pooled data analysis of the effect of CD40 stimulation on levels of p-AKT in co-cultured CLL cells as in (C). (**E**) shows a pooled data analysis of the effect of CD40 stimulation on levels of PTEN in co-cultured CLL cells as in (C).

We have previously shown that CD40 stimulation (achieved by co-culturing CLL cells with CD154-expressing fibroblasts) protected leukemic cells from killing by cytotoxic agents that induce apoptosis through activating the intrinsic mitochondrial or extrinsic death receptor-mediated pathway [34]. Although the cytoprotective effects of CD40 stimulation are known to be largely mediated by the transcription factor NF-κB [11], stimulating CLL cells with soluble CD40 ligand also resulted in activation of AKT, as measured by increased phosphorylation at serine 473 [21, 35, 36]. To establish whether AKT is also activated by membrane-bound CD40 ligand, levels of phospho-AKT (p-AKT) were measured in primary CLL cells cultured on an adherent monolayer of CD154-expressing fibroblasts. As shown in Figure 1A, the level of p-AKT was consistently increased in CLL cells upon CD40 stimulation when compared to cells co-cultured with control parental cells over a period of 72 h. Furthermore, the total AKT in CD40-stimulated cells appeared to be mostly located in a higher molecular weight band (Figure 1A), suggesting that most of it becomes phosphorylated. It was also noted that the level of total AKT was reduced when it was phosphorylated. Since the p-AKT and total AKT were probed on 2 separate membranes, reduction of total AKT is thus likely caused by the accelerated proteasomal degradation of p-AKT that serves as a negative feedback mechanism to terminate AKT activation [37]. To confirm that the CLL cells had been stimulated via CD40, we measured expression of BCL-X_L_ as a surrogate marker of such stimulation [34]. As expected, BCL-X_L_ was up-regulated in CLL cells co-cultured with CD154-expressing fibroblasts throughout the 72 h incubation period (Figure 1A). The pooled densitometry data analysis showed that the increase in p-AKT following CD40 stimulation was maximum at 24 h when levels were 2-fold higher compared with CLL cells that had been co-cultured with the parental fibroblasts (*P* < 0.05) (Figure 1B).

To understand how CD40 stimulation might increase AKT phosphorylation, we investigated its effect on the expression of PTEN (the major negative regulator of PI3K/AKT [38]). Experiments were performed in the presence or absence of interleukin (IL)-4 or IL-21, both of which are produced by T cells and enhance and/or modulate the effect of CD40 stimulation [10, 39]. As expected, CD40 stimulation alone produced an increase in p-AKT (Figure 1C). In contrast, levels of PTEN were markedly decreased in the CLL cells co-cultured with CD154-expressing fibroblasts when compared to that in cells co-cultured with parental fibroblasts (Figure 1C). The analysis of pooled densitometry data showed that this decrease in PTEN was also statistically significant at 24 h (Figure 1E). The increase in p-AKT and decrease in PTEN following CD40 stimulation were both largely unaffected by the addition of IL-4 or IL-21 (Figure 1C–1E). Taken together, these findings clearly demonstrate that CD40 stimulation induces the activation of AKT and that this activation is associated with, and likely caused by, reduced expression of PTEN.

### Inhibition of bendamustine-induced cell death by CD40 stimulation is mediated by AKT

**Figure 2:**
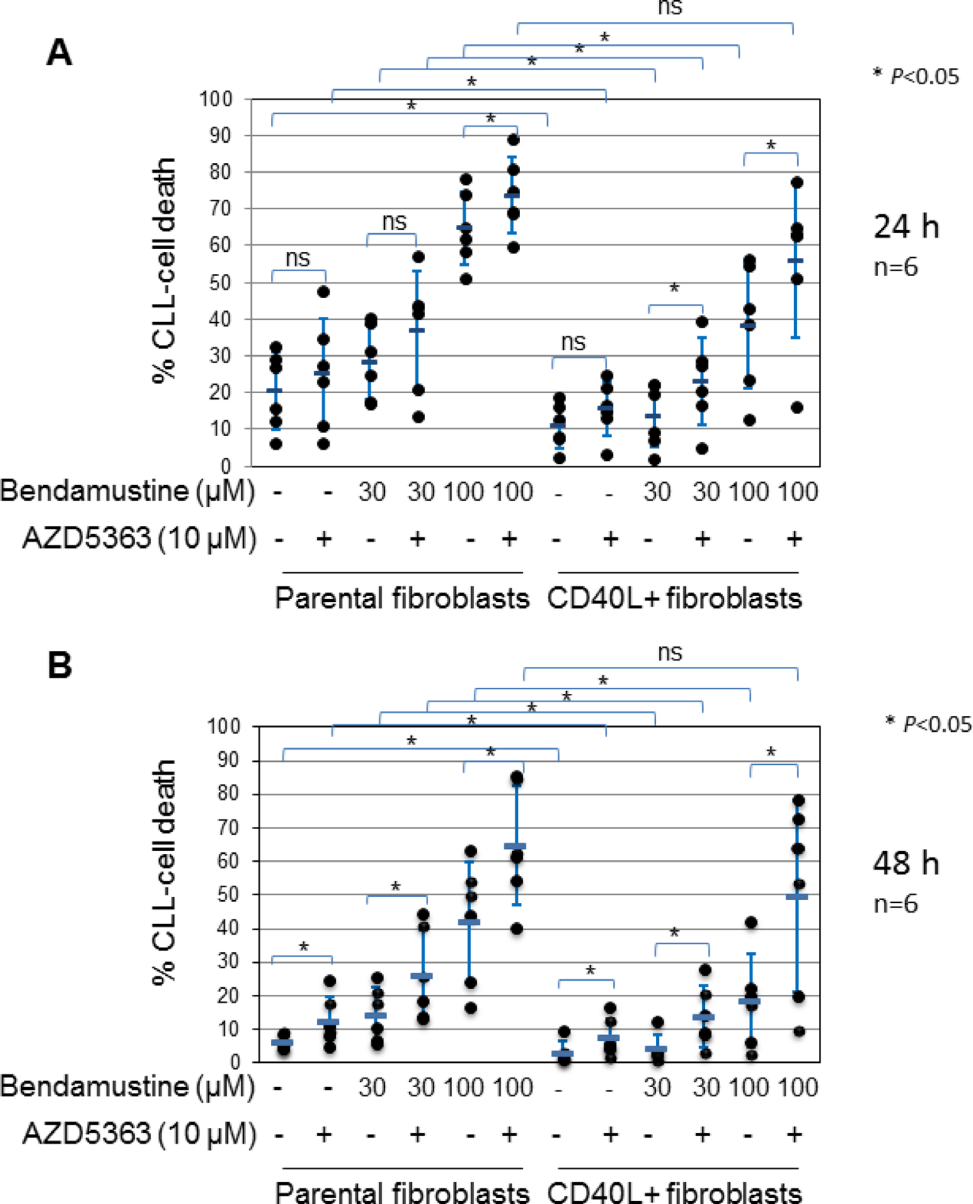
AKT is required for CD40 stimulation-mediated survival and inhibition of bendamustine-induced cell death CLL cells were cultured on a monolayer of control or CD154-expressing fibroblasts in the presence or absence of bendamustine at the indicated concentration with or without AZD5363 (10 μM) for 24 h (**A**) and 48 h (**B**). Co-cultured CLL cells were then harvested at the indicated time points and cell death was determined by PI inclusion on flow cytometry as described in Method. Each data set represents mean ± SD from 6 independent experiments using CLL cells from 4 different patients. Also actual data from individual CLL samples are shown in filled dot.

Having shown that AKT is activated by CD40 stimulation in CLL cells, we next sought to establish whether activated AKT contributes to the cytoprotective effect of CD40 stimulation against drug-induced killing in stimulated cells. To this end, we used a novel, selective ATP-competitive inhibitor of AKT, AZD5363 [40]. Preliminary experiments showed that the inhibitor potently inhibited AKT activity, as measured by de-phosphorylation of GSK3α/β, in CLL cells after incubation for 24 h under standard conditions with an estimated IC_50_ of 3 µM (Supplementary Figure 1A and 1B). However, AZD5363 at concentrations up to 30 µM did not induce cell death in these cells (Supplementary Figure 1C and 1D). The inhibitor also produced a concentration-dependent reduction of AKT activity in CD40-stimulated CLL cells with 10 µM AZD5363 achieving greater than 50% inhibition (Supplementary Figure 2A and 2B). We therefore used AZD5363 at a concentration of 10 µM for subsequent experiments. We chose the cytotoxic drug bendamustine owing to its expanding role in the treatment of previously untreated and recurrent CLL [41, 42]. CLL cells were co-cultured on monolayers of control or CD154-expressing fibroblasts in the presence or absence of bendamustine with or without the AKT inhibitor, and monitored for the induction of cell death at 24 and 48 h. As shown in Figure 2A and 2B, CLL cells co-cultured with CD154-expressing fibroblasts had a reduced spontaneous cell death at both time points when compared with cells co-cultured with control fibroblasts. In addition, cell death induced by bendamustine at both 30 and 100 μM was significantly reduced in CD40-stimulated CLL cells as compared to cells co-cultured with control fibroblasts at 24 and 48 h (Figure 2A and 2B). Importantly, addition of 10 μM AZD5363 not only increased spontaneous cell death in cells co-cultured with CD154-expressing fibroblasts, but also restored sensitivity of CD40-stimulated CLL cells to bendamustine-induced killing to the levels seen in the CLL cells co-cultured with control fibroblasts at both time points (Figure 2A and 2B). The above results clearly indicate that AKT mediates chemoresistance due to CD40 stimulation.

### Stimulation of CLL cells via CD40 + IL-4 or IL-21 induces cell growth that is partly dependent on AKT

We next focused our attention on the growth and proliferation of CLL cells given the established role of AKT in regulating these processes in other cells. We first examined co-cultured CLL cells for an increase in cell size which is known to be one of the functional consequences of CD40 stimulation [43]. To do this, we used forward light scatter (FSC) obtained by flow cytometry as an indicator of cell size, as described [43]. We first compared the FSC properties of CLL cells co-cultured with CD154-expressing versus parental fibroblasts in the presence of IL-4. As expected, a time-dependent increase in FSC was observed in CLL cells co-cultured with CD154-expressing fibroblasts, whereas no such increase was seen in cells co-cultured with control fibroblasts (Supplementary Figure 3A and 3B). The increase in FSC in CD40-stimulated cells was observed as early as day 1 following co-culture with CD154-expressing fibroblasts and continued to increase over the next 3 days (Supplementary Figure 3B). Similar experiments were performed with CLL cells stimulated by CD40 + IL-21 (since IL-21 induces apoptosis in CLL cells cultured in the absence of CD40 stimulation [39], the control for this experiment did not include IL-21). As with CD40 + IL-4, a similar time-dependent increase in FSC was observed in CD40 + IL-21-stimulated cells, whereas no such increase was observed in CLL cells co-cultured with control fibroblasts alone (Supplementary Figures 3C and 3D). Again, the increase in cell size was statistically significant on day 1 following co-culture with CD154-expressing fibroblasts and increased further over the next 3 days (Supplementary Figure 3D).

To determine whether the increased size of CLL cells induced by CD40 stimulation was mediated by AKT, co-cultured CLL cells were again treated with the AKT inhibitor AZD5363. As shown in Supplementary Figure 3E, AZD5363 significantly reduced the increase in cell size due to stimulation by CD154 plus IL-4, although not to the background levels seen in cells co-cultured with control fibroblasts. Similar levels of inhibition of cell expansion by AZD5363 were also observed in CLL cells co-cultured with CD154-expressing fibroblasts in the presence of IL-21 (Supplementary Figure 3F).

To confirm the effects observed with AZD5363, we repeated the experiments using another AKT inhibitor: MK-2206. We chose MK-2206 as it inhibits AKT activity by binding to the plextrin-homology domain of the enzyme, preventing its translocation to the membrane and subsequent activation [44], thus utilising a mode of action different to that of AZD5363. MK-2206 has also been shown to induce apoptosis in CLL cells *in vitro*, with an LC_50_ of 8 µM after 72 hours incubation under standard culture conditions [45]. We therefore used MK-2206 at a concentration of 1, 3 and 10 µM in our experiments. Similar to AZD5363, MK-2206 inhibited the CD40 stimulation-induced increase in cell size in a concentration-dependent manner in all CLL samples examined (Supplementary Figure 3G). Taken together, the above results strongly suggest that increase in size of CLL cells induced by CD40 stimulation is at least in part mediated by AKT.

### CLL cells undergo mitosis following stimulation by CD40 in the presence of IL-4 or IL-21

**Figure 3:**
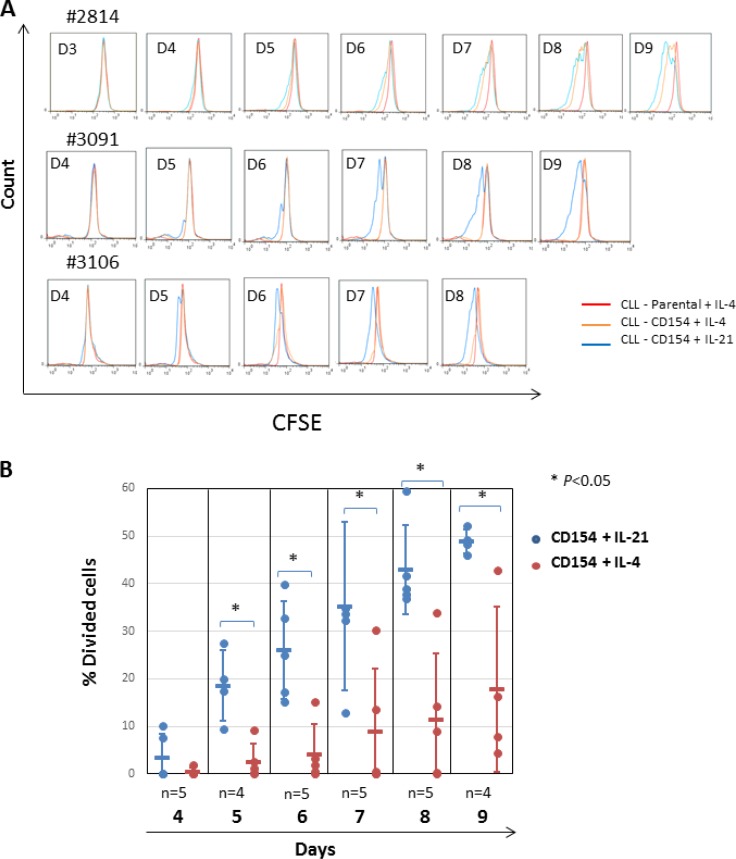
Stimulation by CD154 + IL-21 is more potent than that of CD154 + IL-4 in inducing proliferation of CLL cells *in vitro* (**A**) CFSE labelled CLL cells were co-cultured with CD154-expressing fibroblasts in the presence of recombinant human IL-4 (10 ng/ml) or IL-21 (12.5 ng/ml) over a period of up to ten days. CFSE labelled CLL cells co-cultured with parental fibroblasts were used as a negative control. At the indicated time points, co-cultured CLL cells were harvested and proliferation was measured by reduction of CFSE fluorescence intensity on flow cytometry as described in Method. Three representative CFSE histograms from the time-course experiments using primary CLL cells from 5 different samples are shown. (**B**) Pooled data analysis of the divided cell population among CLL cells co-cultured as in (A) shows that stimulation of CD40 + IL-21 induced significantly higher percentage of divided cells than that of CD40 + IL-4. Percentage of divided cells was calculated using FlowJo software as described in Method.

We next examined the role of AKT in CLL-cell mitosis. To do this, CLL cells pre-incubated with the supravital fluorochrome CFSE were co-cultured with CD154-expressing fibroblasts in the presence of recombinant human IL-4 or IL-21. Proliferation was detected as a reduction in fluorescence intensity due to CFSE as described previously [39]. CFSE-labelled CLL cells were also co-cultured with parental fibroblasts as a negative control. As shown in Figure 3A, CLL-cell proliferation was more rapid, pronounced and consistent following stimulation with CD154 + IL-21 as compared with CD154 + IL-4 (Figure 3A). To establish the identity of the divided cells, aliquots of cells harvested at the indicated time points were tested for expression of CD5 and CD19. As shown in Supplementary Figure 4, most of the cells with reduced CFSE fluorescence intensity were shown to be double positive for both CD5 and CD19, confirming that these divided cells were indeed CLL cells.

Quantitative analysis of the percentage of divided cells in the time-course experiments confirmed that CD154 + IL-21 was more potent than CD154 + IL-4 in inducing CLL-cell mitosis in terms of both magnitude and speed (Figure 3B). Thus, CD154 + IL-21 produced a significantly higher percentage of divided cells, with a significant difference seen as early as day 5 and persisting for at least 9 days (Figure 3B).

### AKT contributes to mitosis induced by CD40 + IL-4

**Figure 4:**
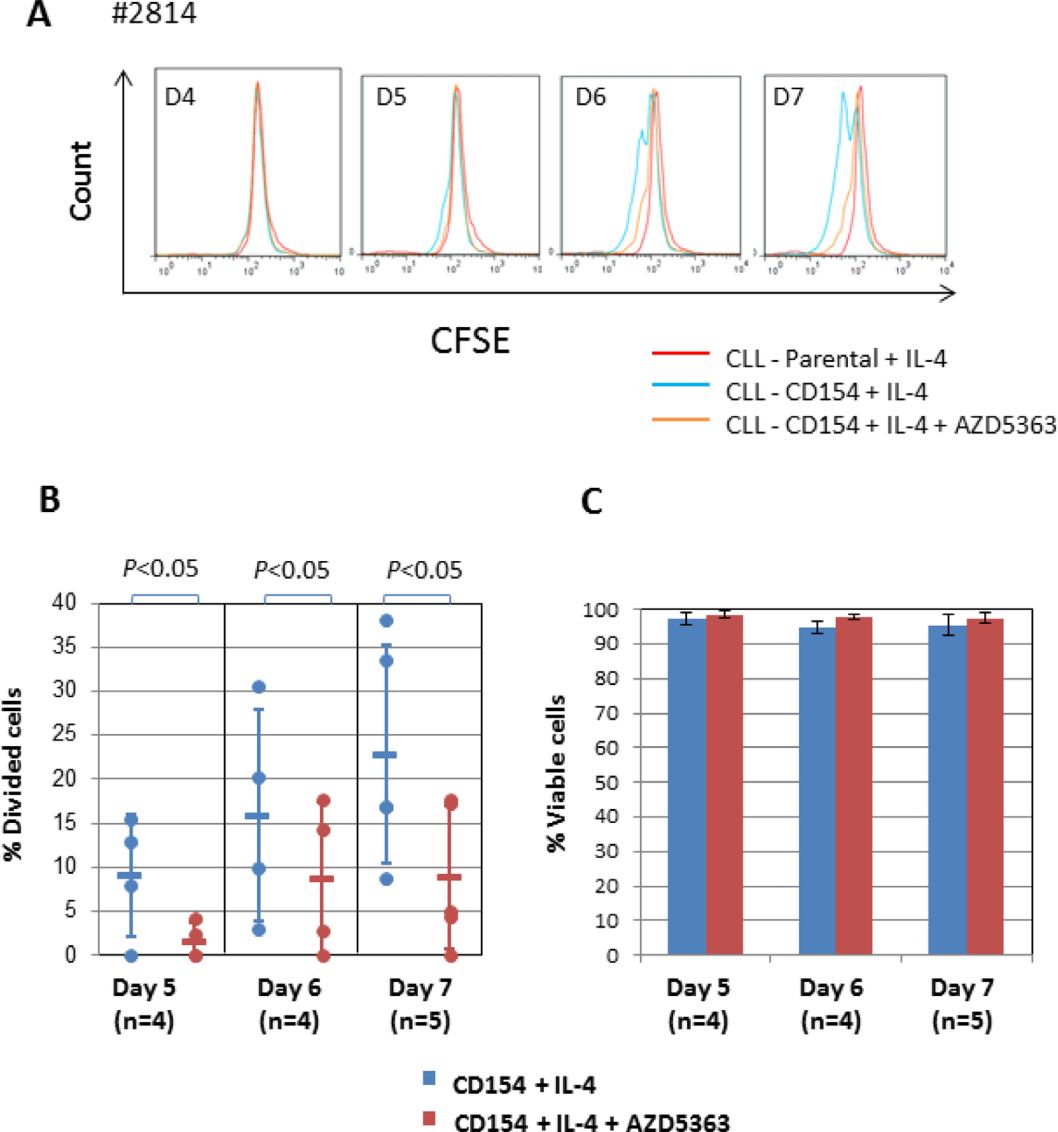
AKT is required for proliferation induced by CD154 + IL-4 (**A**) CFSE labelled CLL cells were co-cultured with CD154-expressing fibroblasts in the presence of recombinant human IL-4 (10 ng/ml) in the presence or absence of AKT inhibitor AZD5363 (10 µM) over a period of 7 days. CFSE labelled CLL cells co-cultured with parental control fibroblasts were used as a negative control. At the indicated time points, co-cultured CLL cells were harvested and measured for reduction of CFSE fluorescence intensity by flow cytometry as in Figure 3A. Representative CFSE histograms of co-cultured CLL cells between 4 and 7 days from one of the five patient samples are shown. (**B**) Pooled data analysis of % divided cells among co-cultured CLL cells shows that addition of AZD5363 (10 µM) significantly reduced % divided cells in CD40 + IL-4-stimulated CLL cells. (**C**) Viability of co-cultured CLL cells as in (B) was determined by PI exclusion/flow cytometry method as described in Method. Pooled data analysis shows that 10 µM AZD5363 had no effect on cell viability of co-cultured cells.

Next, we examined the role of AKT in CLL-cell proliferation induced by CD154 + IL-4. To do this, we again used AZD5363 at 10 µM. As shown in Figure 4A, treatment of the stimulated CLL cells with AZD5363 significantly inhibited the loss of fluorescence due to CFSE, indicating that AKT contributes to mitosis induced by CD154 + IL-4. This was confirmed by quantitative analysis (Figure 4B). Importantly, AZD5363 had no effect on the viability of CLL cells stimulated by CD154 + IL-4 (Figure 4C). This observation indicates that the anti-mitotic effect of AZD5363 was specific and not simply the consequence of cytotoxicity. It also suggests that IL-4 can overcome the pro-death effects of AKT inhibition in the CD154 co-culture system.

### AKT contributes to mitosis induced by CD40 + IL-21 in most but not all cases

**Figure 5:**
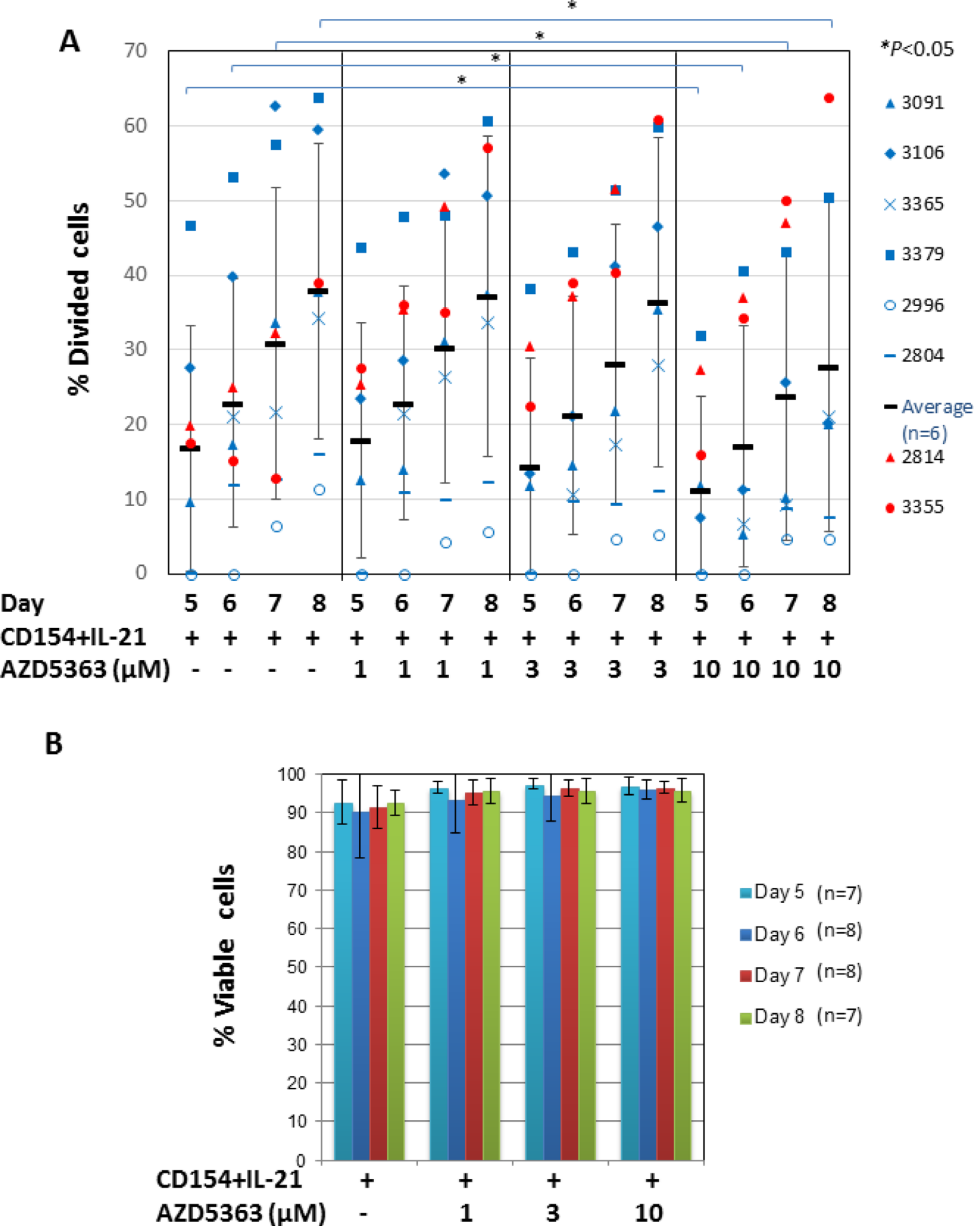
AKT is only required for CD154 + IL-21-induced proliferation in CLL cells from most, but not all patients (**A**) CFSE labelled CLL cells were co-cultured with CD154-expressing fibroblasts in the presence of recombinant human IL-21 (12.5 ng/ml) in the presence or absence of AZD5363 at the indicated concentrations over a period of 8 days. At the indicated time points, co-cultured CLL cells from eight individual patient samples were harvested and measured for reduction of CFSE fluorescence intensity by flow cytometry as in Figure 3A. Percentage of divided cells was calculated as in Figure 3B. Data from samples that were responsive to inhibition of proliferation by AZD5363 as indicated by blue symbols is represented by mean ± SD from independent experiments using CLL cells from six different patients. Data from samples where proliferation was not inhibited by AZD5363 is indicated with red symbols. (**B**) Viability of co-cultured CLL cells as in (A) was determined as in Figure 4C. Pooled data analysis shows that AZD5363 at the indicated concentrations had no effect on cell viability of co-cultured cells as described in (A).

We then investigated the role of AKT in CLL-cell proliferation induced by CD154 + IL-21 using a similar approach, but employing a range of concentrations of the inhibitor. In contrast to the results obtained with CD154 + IL-4, pooled analysis of eight CLL samples stimulated with CD154 + IL-21 failed to show any significant changes in the percentage of divided cells as a result of AKT inhibition with AZD5363 (*P* > 0.05). However, analysis of individual patient samples showed that the AKT inhibitor reduced the percentage of divided CLL cells in a concentration-dependent manner in six out of the eight cases (Figure 5A, blue symbols). AZD5363 at 10 µM concentration has significantly reduced the percentage of proliferating CLL cells in these samples at days 5, 6, 7 and 8 (*P* < 0.05, Figure 5A). In contrast, AZD5363 failed to inhibit proliferation induced by CD154 + IL-21 in two other CLL samples (Figure 5A, red symbols). In the case of sample 3355, the AKT inhibitor further increased proliferation induced by CD154 + IL-21. AZD5363 at these concentrations did not induce cell death in CLL cells from all patient samples (Figure 5B), indicating that its effect on proliferation was selective and not due to cytotoxicity. It also suggests that IL-21 can overcome the pro-death effects of AKT inhibition in the CD154 co-culture system.

To confirm the anti-proliferative effects of AZD5363, the above experiments were repeated using MK-2206 at concentrations of 1, 3 and 10 µM. In the six samples where proliferation was inhibited by AZD5363, MK-2206 produced a concentration-dependent inhibition of CLL-cell mitosis induced by CD154 + IL-21 (Supplementary Figure 5A, blue symbols). MK-2206 at concentrations of 1 and 3 µM had no effect on cell viability at these concentrations (Supplementary Figure 5B). Similarly, in the two samples where proliferation was not inhibited by AZD5363, MK-2206 at concentrations of 1 and 3 µM did not significantly inhibit CLL-cell mitosis induced by CD154 + IL-21 (Supplementary Figure 5A, red symbols). When MK-2206 was used at a concentration of 10 µM, a reduction in the percentage of divided cells was observed (Supplementary Figure 5A). However, this corresponded to a reduction in viability (Supplementary Figure 5B). MK-2206 at 10 µM reduced the viability particularly in the two samples where proliferation was not inhibited by AZD5363 and MK-2206 at 1 and 3 µM concentrations, with an average viability of 51% at day 5, 50% at day 6, 24% at day 7 and 23% at day 8, respectively. This indicates a cytotoxic effect of the inhibitor at this concentration. Taken together, the two AKT inhibitors with different modes of action produced similar effects on CLL-cell mitosis induced by CD154 + IL-21, both inhibiting proliferation in the same six patient samples but not inhibiting such proliferation in the two other samples.

### AKT inhibition in CD40 + IL-21-stimulated cells is associated with repression of cyclin A2 and CDK1

In an attempt to identify molecules involved in the anti-proliferative effect of AKT inhibition, the same CLL samples were co-cultured with CD154-expressing fibroblasts in the presence of IL-21 with or without AZD5363 (10 µM) for 24 h, 48 h and 72 h and analysed by Western blotting for the expression of key molecules involved in cell-cycle regulation. The results were compared between cases that were responsive or unresponsive to the anti-proliferative effects of AKT inhibition. CLL cells co-cultured with the parental fibroblasts were used as a control. First, we compared the expression of cyclins (e.g. A2, D2, D3, E1) and negative regulators of cyclin-dependent kinases (including p27 and p21).

**Figure 6:**
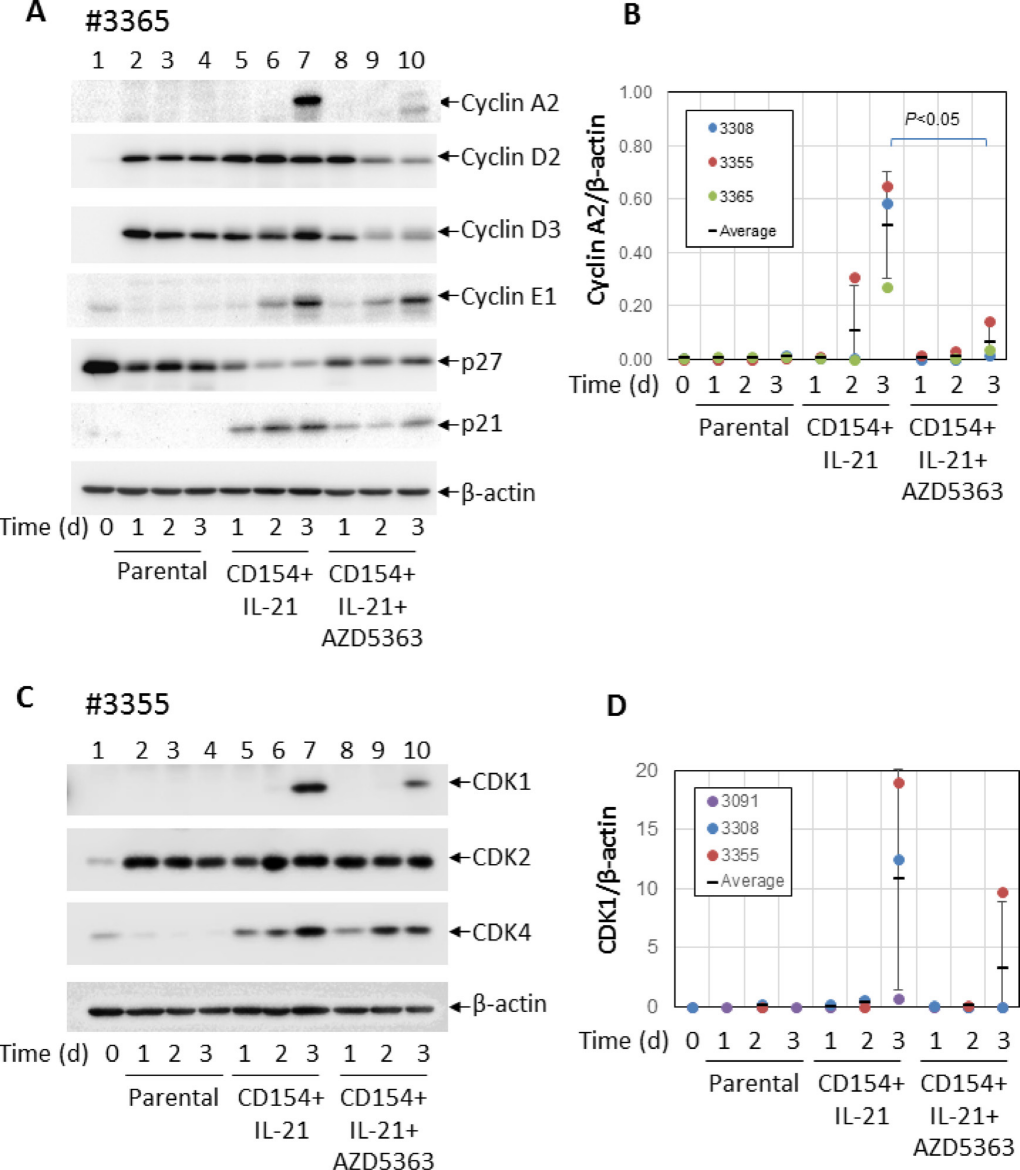
AKT is required for the induction of cyclin A2 and CDK1 by CD40 + IL-21 stimulation (**A**) CLL cells were cultured on a monolayer of control or CD154-expressing fibroblasts in the presence of recombinant human IL-21 (12.5 ng/ml) over a period of 3 days with or without 10 µM AZD5363. At the indicated time points, CLL cells were harvested and analysed for levels of cyclins A2, D2, D3 and E1, and p27 and p21 by Western blotting. β-actin was used as a loading control for densitometric analysis. One representative set of blots from the three of the 5 CLL samples described in Figure 5A is shown. (**B**) shows a pooled data analysis of the effect of AZD5363 on level of cyclin A2 in the co-cultured CLL cells as in (A). (**C**) CLL cells co-cultured under conditions as in (A) were harvested and analysed for levels of CDKs 1, 2 and 4 by Western blotting. β-actin was used as a loading control for densitometric analysis. One representative set of blots from the three of the 5 CLL samples described in Figure 5A is shown. (**D**) shows a pooled data analysis of the effect of AZD5363 on level of CDK1 in the co-cultured CLL cells as in (C).

In keeping with previous reports [46, 47], unstimulated CLL cells expressed higher levels of p27 (Figure 6A, lane 1). Levels of p27 were reduced following co-culture with parental control fibroblasts (Figure 6A lanes 2–4) and decreased further following co-culture with CD154-expressing fibroblasts + IL-21 (Figure 6A, lanes 5–7). We also examined the expression of p21 but failed to detect it in unstimulated CLL cells (Figure 6A, lanes 1–4), although it was detected at low levels in CLL cells co-cultured on CD154-expressing fibroblasts (Figure 6A, lanes 5–7). Addition of AZD5363 restored the expression of p27 but had little effect on the expression of p21 (Figure 6A, lanes 8–10). There was no difference in the expression of p27 and p21 or in the effect of AZD5363 on this expression between CLL samples that were responsive or unresponsive to the anti-proliferative effects of AKT inhibition.

As expected, unstimulated CLL cells expressed hardly any cyclins (Figure 6A, lane 1). Co-culture with control fibroblasts induced the expression of cyclins D2 and D3, but not cyclins A2 and E1 (Figure 6A, lanes 2–4). However, cyclins A2 and E1 were upregulated following stimulation by CD154 + IL-21 (Figure 6A, lanes 5–7). Interestingly, addition of AZD5363 consistently inhibited induction of cyclin A2, but not cyclin E1 in all the samples examined (Figure 6A, lanes 8–10). Pooled data analysis showed that inhibition of cyclin A2 expression by AZD5363 was statistically significant (Figure 6B). There was again no difference in the expression of cyclins or in the effect of AZD5363 on this expression between CLL samples that were responsive or unresponsive to the anti-proliferative effects of AKT inhibition.

Next, we compared expression of cyclin-dependent kinases (CDKs) 1, 2 and 4 in these CLL cells. As shown in Figure 6C (lane 1), unstimulated CLL cells expressed very little amount of these CDKs. CDK2 expression was increased in CLL cells co-cultured with parental fibroblasts, but unaffected by CD40 stimulation (Figure 6B, compare lanes 5–7 to lanes 2–4) or AZD5363 (Figure 6C, lanes 8–10). CDK4 was induced in CLL cells following stimulation by CD154 + IL-21 (Figure 6C, lanes 5–7), as was CDK1 (Figure 6C, lane 7). However, compared to CDK4, induction of CDK1 appeared to be delayed as it was only detected on day 3 in all the CLL samples examined (Figure 6C, lane 7). CDK1 induction was consistently inhibited by AZD5363 (Figure 6C, lane 10), although pooled data analysis failed to show statistical significance (Figure 6D). Again, there was no difference in the expression of CDKs or in the effect of AZD5363 on this expression between CLL samples that were responsive or unresponsive to the anti-proliferative effects of AKT inhibition.

In summary, co-culture of CLL cells with control fibroblasts reduced p27 expression and increased the expression of cyclin D2, cyclin D3 and CDK2. Stimulation with CD154 + IL-21 further reduced the expression of p27 and increased the expression of p21, cyclin A2, cyclin E1, CDK1 and CDK4. AZD5363 inhibited some of these effects (p27 repression and induction of p21, cyclin A2, cyclin D2, cyclin D3 and CDK1) but not others (induction of cyclin E1, CDK2 and CDK4). Importantly, no differences were observed in the expression of any of these proteins or in the effect of AZD5363 on this expression between CLL samples that were responsive or unresponsive to the anti-proliferative effects of AKT inhibition. The molecular mechanisms responsible for the variable effect of AKT inhibition on CLL-cell proliferation induced by CD154 + IL-21 therefore still remain to be elucidated.

### Proliferation of normal B cells induced by CD154 + IL-4 or IL-21 does not involve AKT

**Figure 7:**
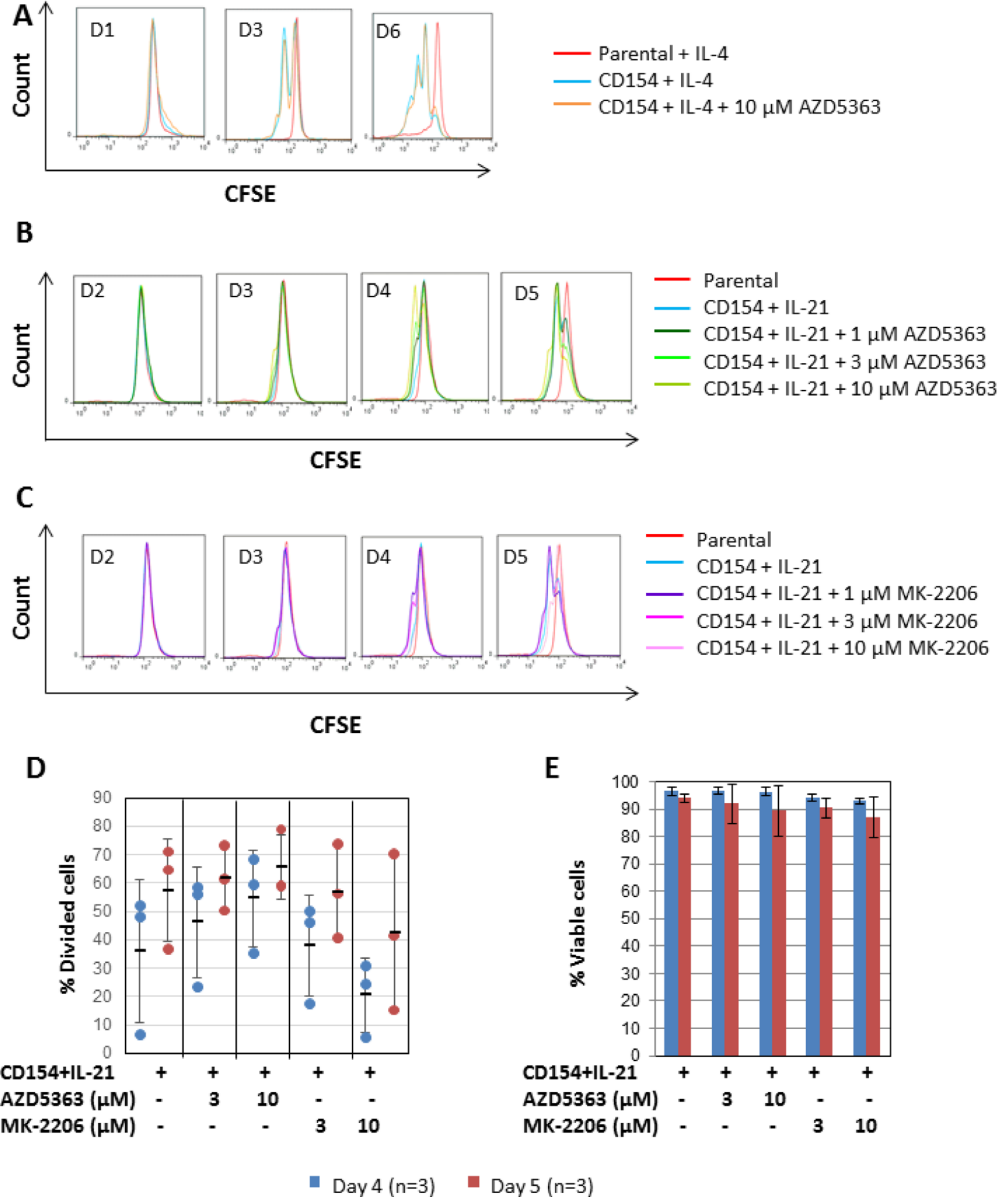
CD40 stimulation-induced proliferation in normal B cells does not require AKT (**A**) CFSE labelled normal B cells were co-cultured with CD154-expressing fibroblasts in the presence of recombinant human IL-4 (10 ng/ml) over a period of six days with or without AZD5363 (10 µM). CFSE labelled normal B cells co-cultured with parental fibroblasts plus IL-4 were used as a negative control. At the indicated time points, co-cultured B cells were harvested and proliferation was measured as in Figure 3A. One set of representative CFSE histograms from the time-course experiments using three individual normal B cell samples is shown. (**B**) CFSE labelled normal B cells were co-cultured with CD154-expressing fibroblasts in the presence of recombinant human IL-21 (12.5 ng/ml) over a period of five days with or without AZD5363 at the indicated concentrations. At the indicated time points, co-cultured B cells were harvested and proliferation was measured as in (A). One set of representative CFSE histograms from three different normal B cell samples examined is shown. (**C**) CFSE labelled normal B cells were co-cultured with CD154-expressing fibroblasts in the presence of recombinant human IL-21 (12.5 ng/ml) over a period of five days with or without MK-2206 at the indicated concentrations. At the indicated time points, co-cultured B cells were harvested and proliferation was measured as in (A). One set of representative CFSE histograms from three different normal B cell samples examined is shown. (**D**) Pooled data analysis shows that neither AZD5363 nor MK-2206 at the indicated concentrations inhibited CD154 + IL-21-induced proliferation in normal B cells. (**E**) Pooled data analysis on the viability of the co-cultured normal B cells as described in (D) shows that neither AZD5363 nor MK-2206 at the indicated concentrations adversely affected the viability of the co-cultured normal B cells.

Finally, to investigate whether inhibition of CD154 + IL-4 or IL-21-induced proliferation by the AKT inhibitors is selective to CLL cells, we repeated the experiment using normal B cells purified from buffy coats by negative selection using a commercially available B cell isolation kit. As shown in Figure 7A, normal B cells were readily induced to proliferate on day 3 when co-cultured with CD154-expressing fibroblasts + IL-4, as indicated by the formation of a sub-peak on the CFSE fluorescence histograms (Figure 7A, *blue line*). In contrast to CLL cells, such proliferation in normal B cells was not inhibited by co-incubation with 10 µM AZD5363 (Figure 7A, *orange line*). This suggests that the anti-proliferative effect of AZD5363 is relatively selective to CLL cells. Consistent with the above observation, normal B cells were also readily induced to proliferate following stimulation by CD154 + IL-21 (Figure 7B, *blue line*). Quantitative analysis of the data showed that significant amounts of divided cells (36% ± 25.1) were detected by day 4 (Figure 7D, *blue dots*). By day 5, % of divided cells increased to 57.5% (±18.2) (Figure 7D, *red dots*). The addition of 1–10 µM AZD5363 had no effect on this proliferation (Figure 7B and 7D). The same was true of 1 – 10 µM MK-2206 (Figure 7C and 7D). Neither AZD5363 nor MK-2206 at the concentrations used in the study induced significant amounts of cell death in stimulated normal B cells (Figure 7E). Collectively, these results suggest that AKT inhibition by AZD5363 or MK-2206 does not inhibit the proliferation of normal B cells induced by CD154 + IL-21 and that AKT is therefore not required for proliferation of normal B cells in response to CD40 stimulation.

## DISCUSSION

This study was conducted to determine whether AKT plays a role in CD40 stimulation-mediated cell survival, growth and proliferation of CLL cells and, in doing so, further elucidate the role of AKT in CLL biology. We confirmed previous reports that CD40 stimulation induces activation of AKT in CLL cells [21, 35, 36]. We also showed that this activation was associated with decreased expression of PTEN, a result consistent with a recent report that CD40 stimulation results in a specific induction of microRNA-22, a cellular inhibitor of PTEN, which subsequently leads to AKT activation in CLL cells [48].

To address the extent to which AKT mediates the pro-survival effect of CD40 stimulation, we co-cultured CLL cells on a monolayer of CD154-expressing fibroblasts and compared their viability to that in cells co-cultured with control fibroblasts. Our demonstration that CD40-stimulated CLL cells underwent less spontaneous cell death and were more resistant to bendamustine-induced killing confirms that CD154 on the surface of the transfected fibroblasts was engaging with CD40 on the CLL cells to generate pro-survival signals. Furthermore, our observations that pharmacological inhibition of AKT reduced the survival of the stimulated CLL cells and sensitized them to killing by bendamustine strongly implicates AKT as an important mediator of CD40-derived pro-survival signals.

We have also shown for the first time that AKT is required for CD40-induced cell growth as measured by an increase in the size of stimulated CLL cells. This is not unexpected since AKT is known to regulate growth and cell size via mTOR and its downstream targets S6K1 and 4EBP1/eIF4E [49], and by stimulating protein synthesis and inhibiting protein degradation [50]. It has also been shown that AKT maintains cell size and survival by increasing mTOR-dependent nutrient uptake, which is required for protein synthesis [51].

Regarding the variation in kinetics of proliferation observed in our co-culture system between CLL cells and normal B cells and between different cases of CLL, our results are entirely in agreement with *in vivo* studies showing that normal B cells proliferate more rapidly (1.5–4.24% per day) [52, 53] than CLL cells (0.08–1.7% per day) [53, 54] and that proliferation kinetics vary widely between different cases of CLL [53, 55]. Importantly, pharmacological inhibition of AKT inhibited the proliferation of CLL cells in most cases irrespective of the rate of mitosis but had no effect on the proliferation of normal B cells. This observation suggests that AKT plays a more prominent role in signalling proliferation induced by CD40 + IL-4 or IL-21 in CLL cells as compared with B cells.

Our data suggest that the role of AKT in CLL-cell proliferation induced via CD40 is both stimulus and case dependent. The pronounced and consistent inhibitory effect of AKT inhibition on CLL-cell proliferation induced by CD154 + IL-4 implies a crucial role for AKT in signalling mitosis in CLL cells under such conditions. This is entirely in keeping with a recent report demonstrating a key role of the PI3K/AKT pathway in CLL-cell proliferation induced by CD154 + IL-4 [48]. It is also in agreement with a previous report implicating AKT as a mediator of proliferation following stimulation of CLL cells with CpG-oligodeoxynucleotides [56]. In contrast to results obtained with CD40 + IL-4, when CLL cells were stimulated to proliferate by CD154 + IL-21, pharmacological inhibition of AKT reduced proliferation in most but not all patient samples indicating an inconsistent role for AKT in signalling proliferation under these circumstances. A recent report that the JAK inhibitor (ruxolitinib) almost completely inhibited CLL-cell proliferation induced by CD154 + IL-21 [39], suggests that the JAK-STAT pathway may dominate over AKT in transmitting proliferation signals under these circumstances.

In order to identify the molecules responsible for mediating the differential effect of AKT inhibition on proliferation induced by CD40 + IL-21, we compared changes in the expression of cell cycle regulatory molecules following stimulation of CLL with CD154 + IL-21 in the presence or absence of AKT inhibitors. It has recently been shown that lenalidomide inhibits CLL-cell proliferation induced by CD154 + IL-4 + IL-10 in a cereblon/p21-dependent manner but independently of p53 [57]. It is also known that levels of p21 protein can be negatively modulated by AKT through direct or indirect phosphorylation [24–26]. As a result, CDKs are liberated and allowed to restore their kinase function in cell cycle progression. We therefore speculated that the differential effect of AKT inhibition on CD40 + IL-21-induced proliferation could be mediated via differential induction of p21. However, we were unable to detect p21 expression in unstimulated CLL cells and could only detect it at low levels following stimulation with CD154 + IL-21. Importantly, AKT inhibition had little effect on the expression of p21 in all the samples examined. Instead, we found that AKT inhibition reduced the expression of cyclin A2 and CDK1 consistently in all the samples examined irrespective of whether their proliferation was inhibited or not. Therefore, the molecular mechanisms underlying the variable effect of AKT inhibition on CLL-cell proliferation induced by CD40 + IL-21 remain to be elucidated.

In summary, we have shown that AKT is an important mediator of CLL-cell survival, growth and chemoresistance induced by CD40 stimulation. We also showed that AKT is required for proliferation of CLL cells in response to CD154 + IL-4 and contributes to proliferation induced by CD154 + IL-21 in CLL cells in most patient samples. From a clinical perspective, the sensitisation of CLL cells to killing by bendamustine that was observed when the AKT inhibitor was added to the CD154 co-culture system provides a strong rationale for combining AKT inhibitors with chemotherapy-based treatment in order to augment cytoreduction at sites of tissue involvement. Furthermore, the anti-proliferative effect of AKT inhibition suggests that AKT inhibitors might delay re-growth of the residual leukemic clone in most patients as post-induction maintenance therapy.

## MATERIALS AND METHODS

### CLL cell and normal B cell preparation

Peripheral blood samples from CLL patients were obtained with informed consent and the approval of the Liverpool Research and Ethics Committee. The clinical details of the patients included for this study are given in Table 1. CLL cells were isolated by centrifugation of blood over Lymphoprep (Axis-Shield PoC AS, Oslo, Norway) and stored in a –150°C freezer prior to use. Normal B cells were purified by negative selection using a B cell isolation kit (Miltenyi Biotech, Bisley, UK) from Buffy coats obtained from the National Blood Service (Liverpool, UK). B-cell purity was determined by CD19 staining and flow cytometry.

**Table 1:** Clinical characteristics of the CLL samples used

Sample number	Sex	Age at diagnosis	Stage (Binet)	WBC (10^9^/l)	IGHV status^¶^	Chromosomal abnormalities	Prior therapy^*^
#1958	f	65	A	215	M	13q-	Y
#2064	m	46	C	120	UM	Tri12	Y
#2096	m	65	A	60	M	17p- & 13q-	N
#2103	m	56	A	125	UM	17p- & 13q-	N
#2263	m	65	B	366	UM	11q- & 13q-	Y
#2521	f	79	B	74	M	Tri12	Y
#2729	m	66	A	37	M	normal	N
#2804	F	nd^#^	A	39.7	M	nd	nd
#2814	m	72	A	196	UM	normal	N
#2996	m	nd	nd	169.2	M	13q-	nd
#3033	m	66	A	41	M	normal	N
#3058	m	69	B	51	UM	normal	N
#3091	m	nd	nd	41.7	UM	11q-	nd
#3106	m	66	C	115	UM	11q- & 13q-	nd
#3129	m	nd	nd	31.8	UM	nd	nd
#3308	m	66	C	nd	UM	nd	nd
#3353	f	75	A	72	M	nd	nd
#3355	m	73	B	130	M	13q-	nd
#3365	f	nd	C	253	nd	13q-	nd
#3379	m	67	B	228.5	UM	17p-	Y

^¶^IGHV status refers to somatic mutation in IGHV gene of CLL cells as compared with the gene sequence of the nearest germ-line using 2% as a cut-off. M=mutated; UM=un-mutated.

^*^Prior therapy consisted of various combinations of glucocorticoid, chlorambucil, fludarabine, or fludarabine plus cyclophosphamide.

^#^nd: not done/unknown

### Chemicals, antibodies and other reagents

AKT inhibitor AZD5363 was provided by AstraZeneca (London, UK). Another AKT inhibitor MK-2206 was purchased from Selleck Chemical (via Stratech Scientific Ltd., Suffolk, UK). Bendamustine hydrochloride hydrate was obtained from Sigma-Aldrich (Gillingham, UK). Rabbit monoclonal antibodies against phospho-AKT (Ser473) (clone D9E), phospho-GSK-3α/β (Ser21/9) (clone D17D2), CDK2 (clone 78B2), CDK4 (clone D9G3E), Cyclin D2 (clone D52F9), p21 (DCS60) and p27 (D69C12), and mouse monoclonal antibodies to CDK1 (clone POH1), Cyclin A2 (clone BF683), Cyclin D3 (clone DCS22), Cyclin E1 (clone HE12), and rabbit polyclonal antibodies to total AKT, BCL-XL and PTEN were all obtained from Cell Signalling Technology (via New England Biolabs, Herts, UK). Mouse monoclonal antibody to GSK3α/β (clone 21A) was obtained from Life Technologies (Paisley, UK). Mouse monoclonal antibody to β-actin (clone AC-74) was from Sigma-Aldrich. Secondary antibodies, goat anti-mouse IgG-HRP and goat anti-rabbit IgG-HRP were from Santa Cruz Biotechnology (Insight Biotechnology, Middlesex, UK). Other chemicals, unless otherwise stated, were all obtained from Sigma-Aldrich.

### Co-culture of CLL cells

Stably transfected mouse fibroblasts expressing human CD154 and control parental fibroblasts (both kindly provided by Professor Gerald Cohen at University of Liverpool) were maintained as described [34]. After thawing, CLL cells were maintained in RPMI 1640 medium supplemented with 10% heat-inactivated fetal bovine serum, 2 mM L-glutamine, 100 U/ml penicillin, and 100 μg/ml streptomycin (Life Technologies). For CD40 stimulation, CLL cells were seeded on an adherent monolayer of CD154-expressing or control fibroblasts at a ratio of 10:1 and incubated at 37°C for an appropriate period of time, as described [34]. To determine the effects of bendamustine and AZD5363 on cell survival following CD40 stimulation, CLL cells were co-cultured for 24 h or 48 h with the CD154-expressing or parental fibroblasts in the presence or absence of the above drugs before harvesting. To induce proliferation and determine the effect of AKT inhibition on proliferation, CLL cells were co-cultured with CD154-expressing fibroblasts in the presence of recombinant human interleukin-4 (IL-4, R&D Systems, Minneapolis, MN ) or IL-21 (Life Technologies), with or without the AKT inhibitors for up to ten days.

### Analysis of cell death by flow cytometry

CLL cells were co-cultured with CD154-expressing or control fibroblasts in the presence or absence of cytotoxic agents at the indicated concentrations. At the end of the incubation period, co-cultured CLL cells were harvested as described [34] and their viability was determined by propidium iodide exclusion and flow cytometry as previously described [58]. Aliquots of cells from the same experiment were analysed by Western blotting for the expression of specific proteins.

### Analysis of proliferation by flow cytometry

CLL cells were first incubated at 37°C for 10 minutes in complete RPMI 1640 medium containing 0.5 µM carboxyfluorescein diacetate succinimidyl ester dye (CFSE, Molecular Probes^®^, Life Technologies) and then washed with fresh medium to remove excess dye. Cells were then incubated for a further 20 minutes at 37°C and washed again before being re-suspended in fresh medium containing recombinant human IL-4 (at 10 ng/ml) or IL-21 (at 12.5 ng/ml) and seeded onto an adherent monolayer of CD154-expressing fibroblasts described above. At the end of indicated incubation periods, cells were gently removed from the respective monolayers and analysed by flow cytometry using excitation and emission wavelengths of 488 nm and 517 nm, respectively, according to manufacturer’s protocol. FlowJo software (7.6.5 version) (FlowJo, Ashland, OR) was used to analyse proliferation data of CFSE-labelled CLL cells and calculate the percentage of divided cells, as described [39].

### Western blotting

At the end of the incubation, co-cultured CLL cells were harvested and washed once in ice-cold phosphate-buffered saline (PBS) and lysed using 100 μl of RIPA buffer (50 mM Tris/HCl (pH 7.6), 1% (w/v) Triton-X100, 150 mM NaCl, 0.1% (w/v) sodium dodecyl sulfate (SDS), 0.5% (w/v) sodium deoxycholate, and protease and phosphatase inhibitor cocktails (Sigma-Aldrich). Cell lysates were further sonicated using Diagenode Bioruptor® (Diagenode Inc, Liège, Belgium). Sonicated cell lysates were cleared of debris by centrifuging at 10,000g for 10 min at 4°C and protein concentration determined using RC DC Protein Assay Reagents Kit (BioRad laboratories, Herts, UK). SDS polyacrylamide gel electrophoresis (SDS-PAGE) and immunoblotting were performed essentially as described [31]. Briefly, cellular proteins were separated on a SDS-polyacrylamide gel and transferred to Immobilon-P polyvinilidene difluoride membranes (Millipore Corporation, Bedford, MA) which were then probed with the appropriate primary antibodies. Immunoreactivity was detected using the relevant HRP-labelled secondary antibodies (Santa Cruz Biotechnology), which were visualized on an Image Reader LAS-1000 (Fujifilm, Tokyo, Japan) using an enhanced chemiluminescence kit (Millipore). For quantification of the signals, the images were further analysed on the same instrument using 2D Densitometry Aida Image Analyzer software (Fujifilm).

### Statistical analysis

Where appropriate, a two-tailed, paired *t*-test was performed to determine the statistical significance of the difference between the two groups of data.

## SUPPLEMENTARY MATERIALS FIGURES


