# Supplementary material for: Delineating the distinct role of AKT in mediating cell survival and proliferation induced by CD154 and IL-4/IL-21 in chronic lymphocytic leukemia
Source: Oncotarget. 2017 Nov 7;8(61):102948–64. doi: 10.18632/oncotarget.22292 (PMC5732702; doi:10.18632/oncotarget.22292)
Supplement: Supplementary file 1 [file oncotarget-08-102948-s001.pdf]

Delineating the distinct role of AKT in mediating cell survival and proliferation induced by CD154 and IL-4/IL-21 in chronic lymphocytic leukemia

SUPPLEMENTARY MATERIALS

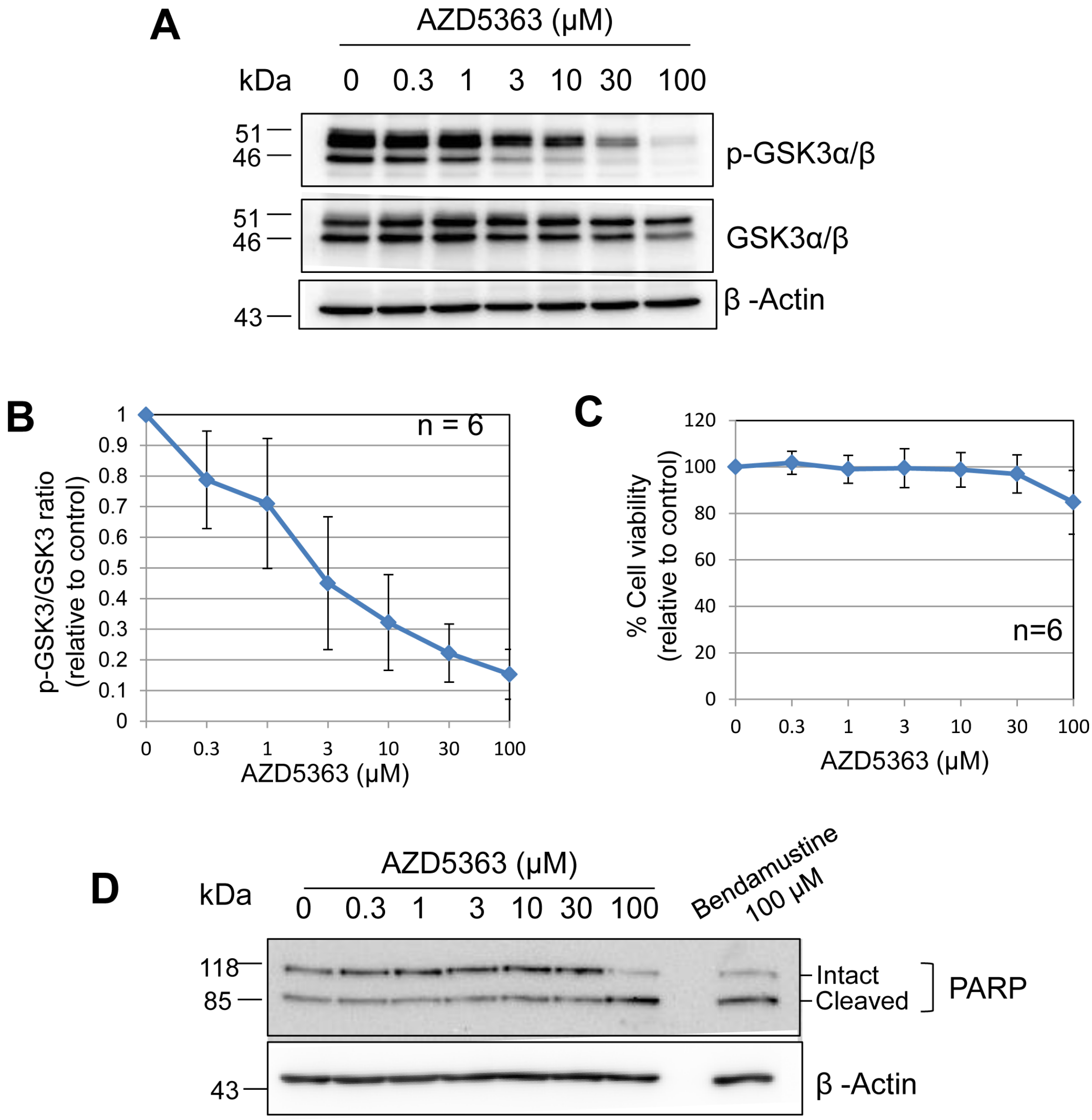

Supplementary Figure 1: AKT inhibitor AZD5363 inhibits AKT activity in primary CLL cells under standard culture condition. (**A**) Primary CLL cells were incubated for 24 h with AZD5363 at the indicated concentrations and then examined by Western blotting for levels of phosphorylated GSK3α/β as a measure of AKT activity. Total GSK3α/β and β-actin were used as loading controls. One representative blot from 6 CLL samples examined is shown. (**B**) Densitometry analysis of the signals corresponding to phosphorylated and total GSK3α/β in CLL cells was performed to quantify the effect of AZD5363 on AKT activity. (**C**) Following incubation with AZD5363 as in A, viability of CLL cells was measured by propidium iodide (PI) exclusion and flow cytometry. (**D**) A representative blot of PARP cleavage in CLL cells treated with AZD5363 as in A is shown. PARP cleavage induced by bendamustine in CLL cells was used as a positive control.

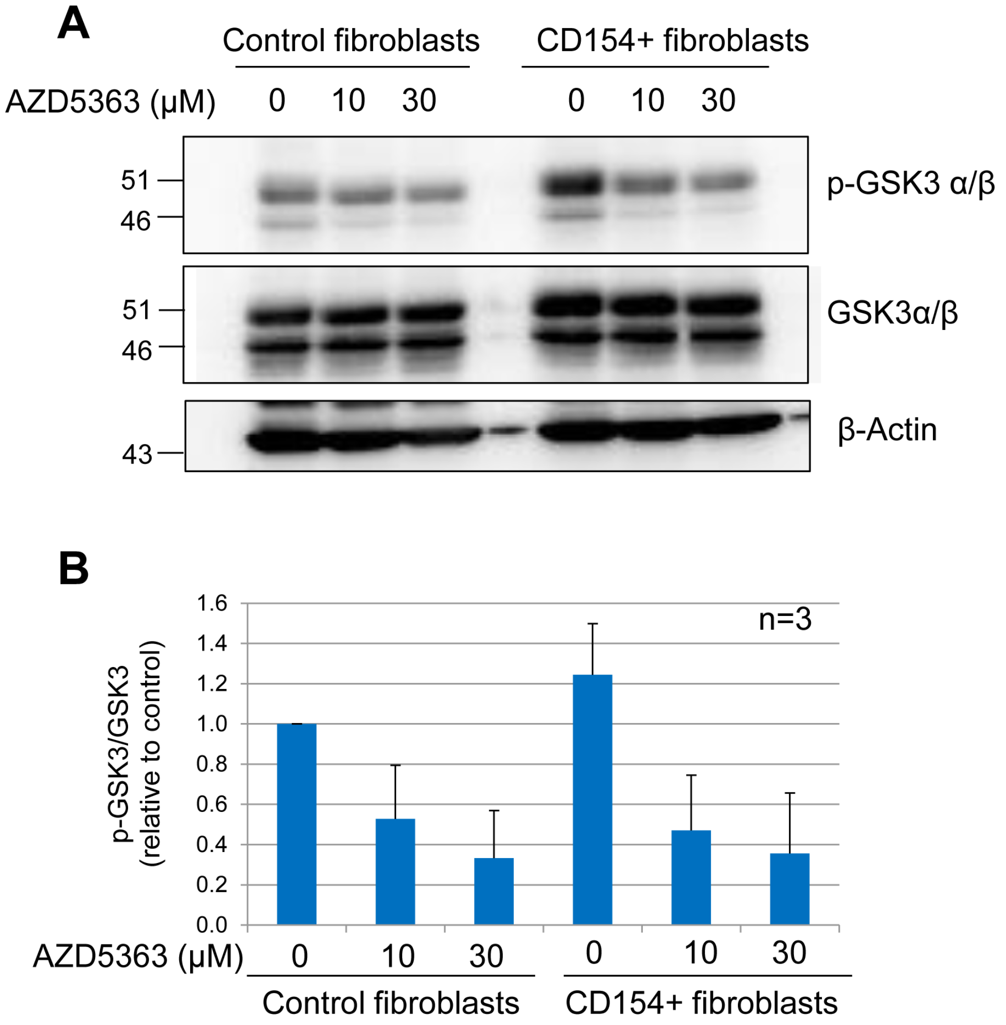

Supplementary Figure 2: FAKT inhibitor AZD5363 inhibits AKT activity in CD40-stimulated CLL cells. (**A**) CLL cells were cultured on a monolayer of control or CD154-expressing fibroblasts for 24 h in the absence or presence of AZD5363 at the indicated concentration. At the end of incubation, co-cultured CLL cells were harvested and analysed for expression levels of p-GSK and total GSK by Western blotting. β-actin was probed as a loading control. One representative blot from 3 CLL samples examined is shown. (**B**) shows a pooled densitometry data analysis of the effect of AZD5363 on levels of p-GSK in co-cultured CLL cells. In this graph, each bar represents the mean ± SD.

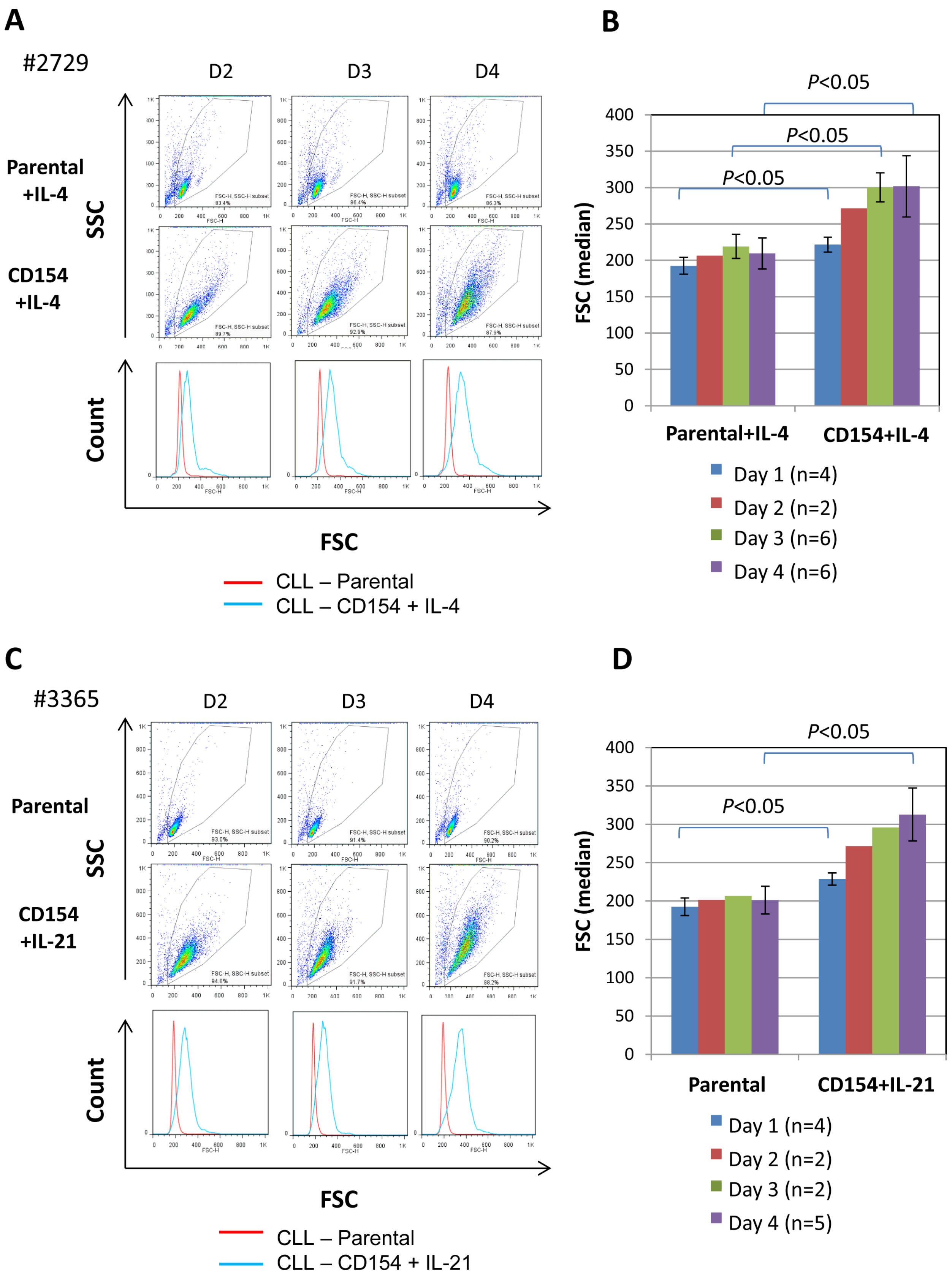
A
#2729
D2
D3
D4
Parental
+IL-4
CD154
+IL-4
SSC
Count
FSC
CLL – Parental
CLL – CD154 + IL-4
B
P<0.05
P<0.05
P<0.05
FSC (median)
400
350
300
250
200
150
100
50
0
Parental+IL-4
CD154+IL-4
Day 1 (n=4)
Day 2 (n=2)
Day 3 (n=6)
Day 4 (n=6)
C
#3365
D2
D3
D4
Parental
CD154
+IL-21
SSC
Count
FSC
CLL – Parental
CLL – CD154 + IL-21
D
P<0.05
P<0.05
FSC (median)
400
350
300
250
200
150
100
50
0
Parental
CD154+IL-21
Day 1 (n=4)
Day 2 (n=2)
Day 3 (n=2)
Day 4 (n=5)

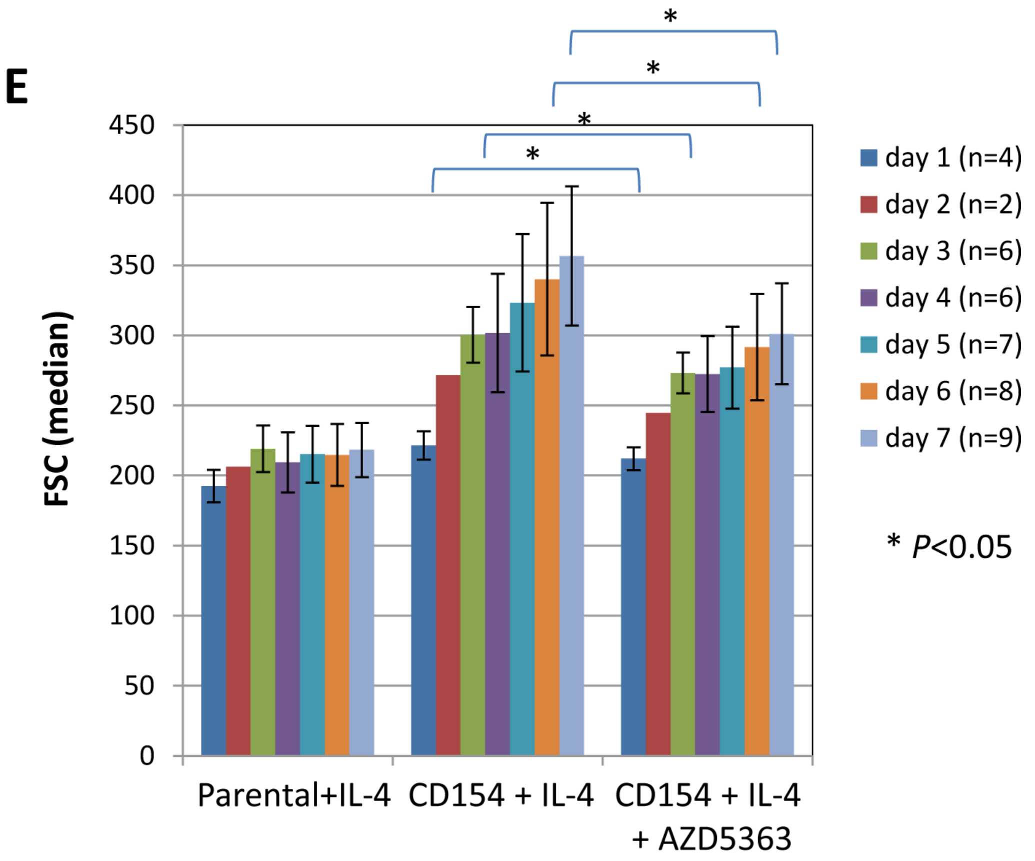

E
FSC (median)
450
400
350
300
250
200
150
100
50
0
Parental+IL-4
CD154 + IL-4
CD154 + IL-4 + AZD5363
day 1 (n=4)
day 2 (n=2)
day 3 (n=6)
day 4 (n=6)
day 5 (n=7)
day 6 (n=8)
day 7 (n=9)
* P<0.05

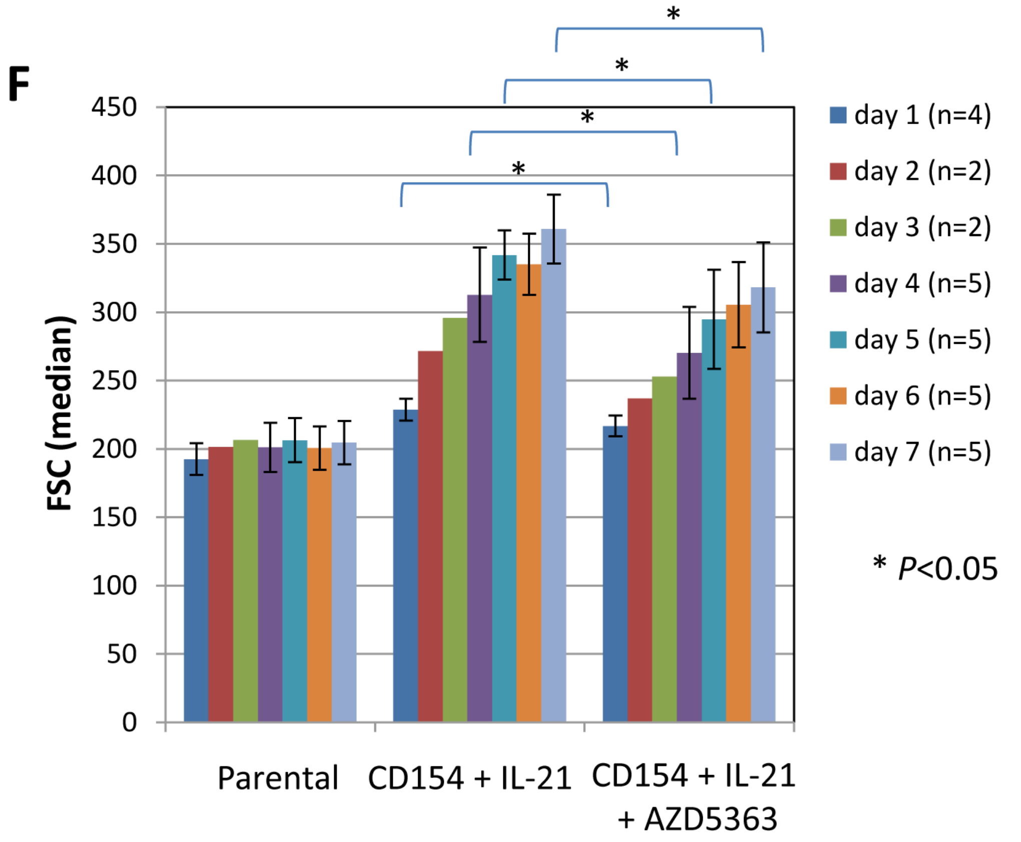

F
FSC (median)
450
400
350
300
250
200
150
100
50
0
Parental
CD154 + IL-21
CD154 + IL-21 + AZD5363
day 1 (n=4)
day 2 (n=2)
day 3 (n=2)
day 4 (n=5)
day 5 (n=5)
day 6 (n=5)
day 7 (n=5)
* P<0.05

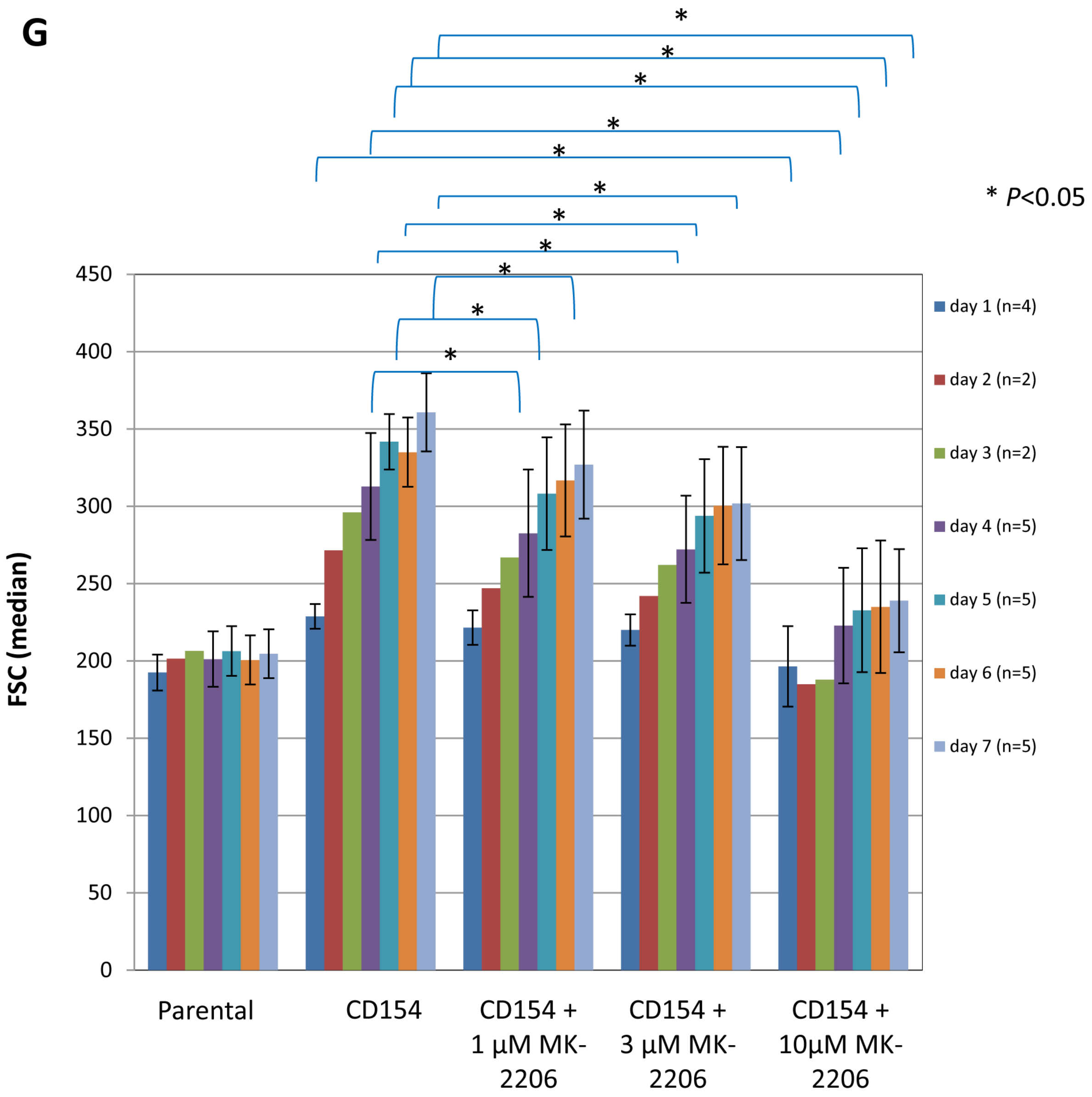

Supplementary Figure 3: AKT inhibition reduces CD40 stimulation-induced cell size increase in CLL cells. (**A**) CLL cells were cultured on a monolayer of parental or CD154-expressing fibroblasts in the presence of recombinant human IL-4 (10 ng/ml) for the indicated times. At the end of incubation, CLL cells were harvested and analysed for cell size according to their forward light scatter (FSC) properties on a flow cytometer. Representative cytograms depicting side scatter (SSC) versus FSC, and FSC histograms of co-cultured CLL cells from one patient are shown. (**B**) Pooled analysis of median FSC of CLL cells co-cultured as in (A) showed a time-dependent increase in cell size in response to CD40 stimulation. (**C**) CLL cells were co-cultured as in (A) in the presence of recombinant human IL-21 (12.5 ng/ml) for the indicated times. At the end of incubation, CLL cells were harvested and analysed for cell size by flow cytometry as in (A). Representative cytograms are shown. (**D**) Pooled analysis of median FSC of CLL cells co-cultured as in (C) showed a time-dependent increase in cell size in response to CD40 stimulation. (**E**) AKT inhibitor (AZD5363, 10 µM) inhibited the increase in cell size of CLL cells induced by CD40 + IL-4 stimulation. (**F**) AKT inhibitor (AZD5363, 10 µM) inhibited the increase in cell size of CLL cells induced by CD40 + IL-21 stimulation. (**G**) AKT inhibitor (MK-2206) inhibited the increase in cell size of CLL cells induced by CD40 + IL-21 stimulation in a time- and concentration-dependent manner.

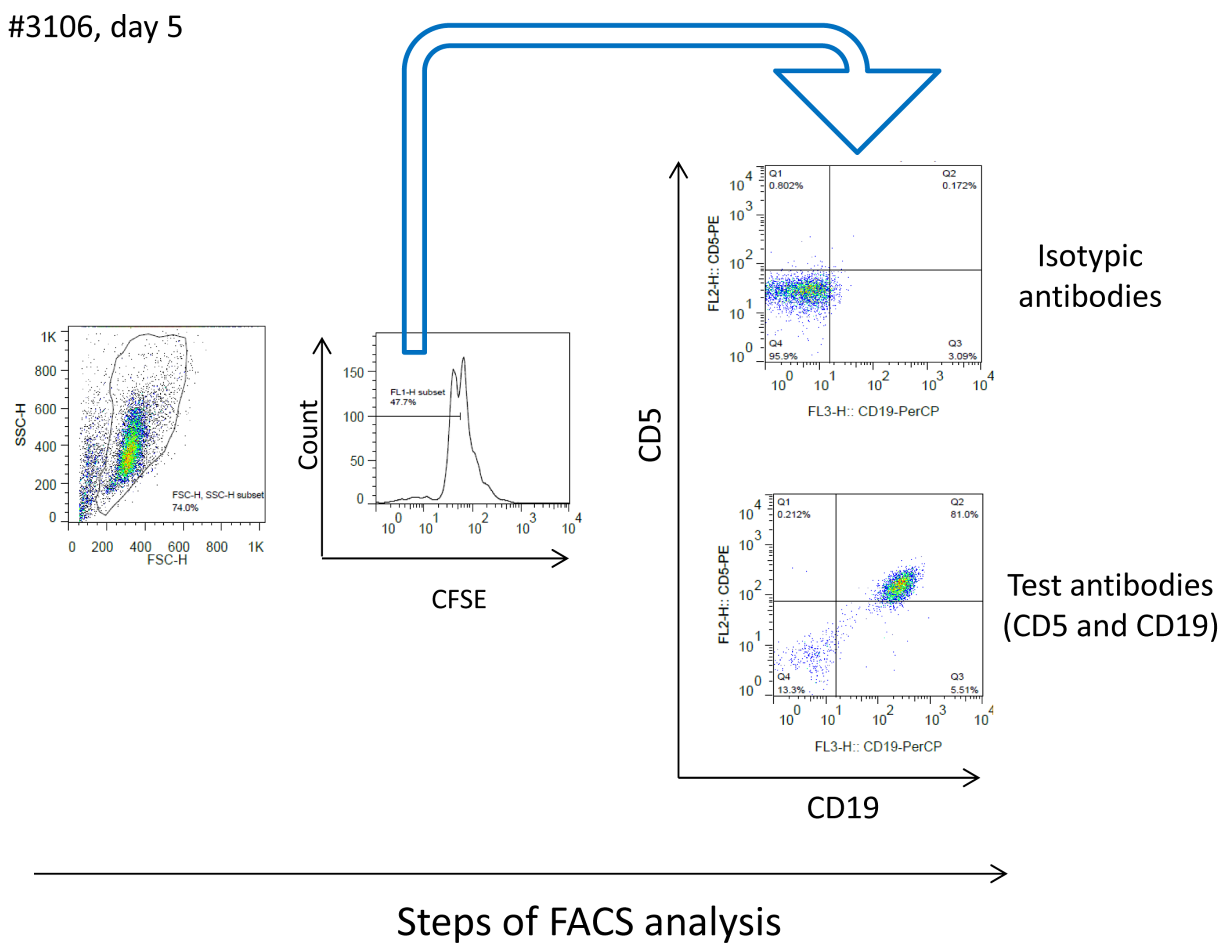

Supplementary Figure 4: Workflow of monitoring CD5^{+} & CD19^{+} double positive CLL-cell population from the divided cells as defined by reduction in CFSE fluorescence on FACS.

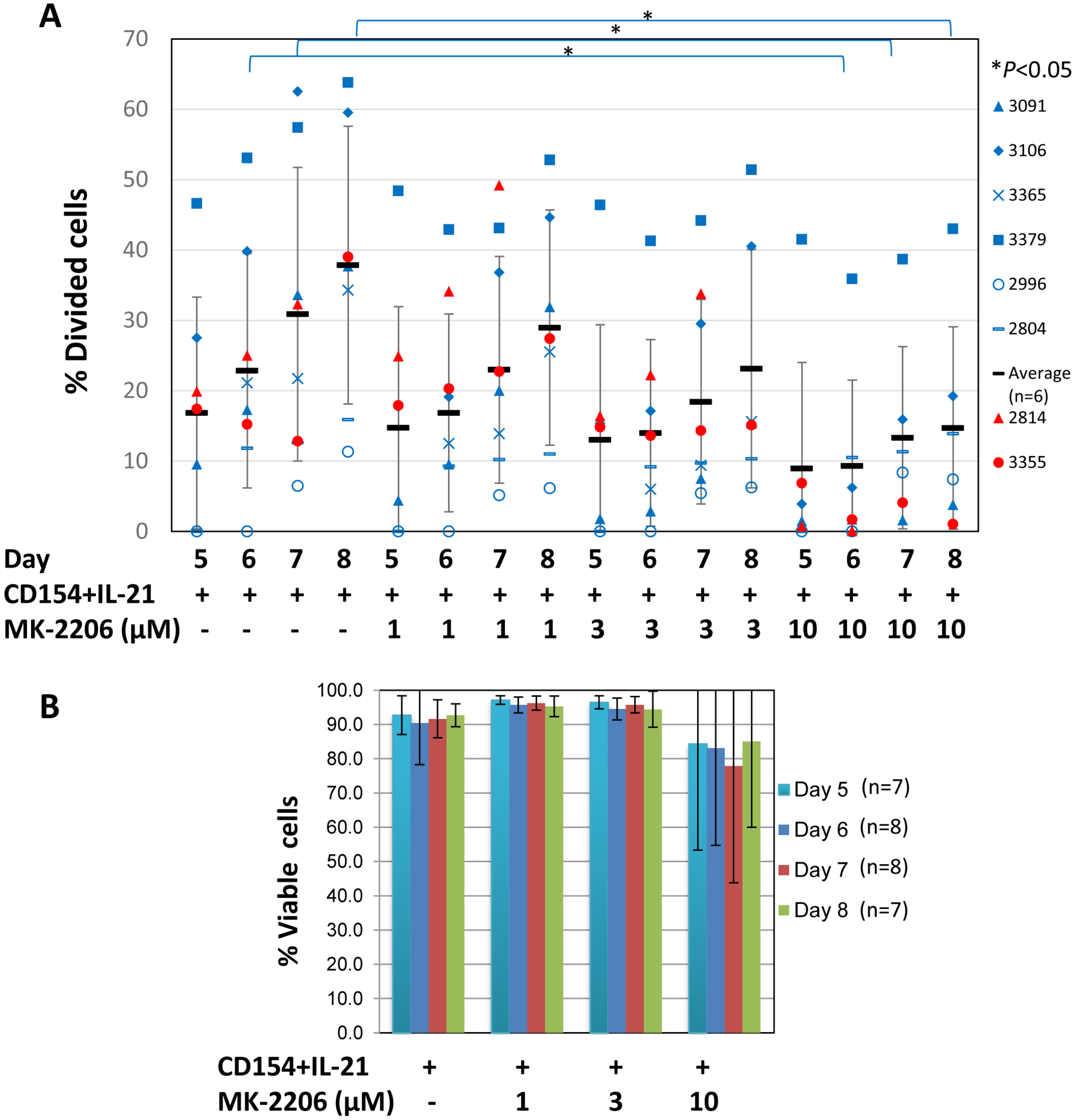

Supplementary Figure 5: AKT is required for CD154+ IL-21-induced proliferation in CLL cells from some but not all patients. (**A**) CFSE labelled CLL cells were co-cultured with CD154-expressing fibroblasts in the presence of recombinant human IL-21 (12.5 ng/ml) in the presence or absence of MK-2206 at the indicated concentrations over a period of 8 days. Proliferation was measured as described in Figure 3A and percentage of divided cells calculated as in Figure 3B. Data from samples that were responsive to inhibition of proliferation by MK-2206 as indicated by blue symbols is represented by mean ± SD from independent experiments using CLL cells from six different patients. Data from samples where proliferation was not inhibited by MK-2206 is indicated with red symbols. (**B**) Viability of co-cultured CLL cells as in (A) was determined as in Figure 4C. Pooled data analysis showed that MK-2206 at 1 and 3 µM concentrations had no effect on cell viability of co-cultured cells. The cell viability of co-cultured cells treated with MK-2206 (10 µM) was reduced, even though the reduction was not statistically significant.
